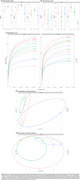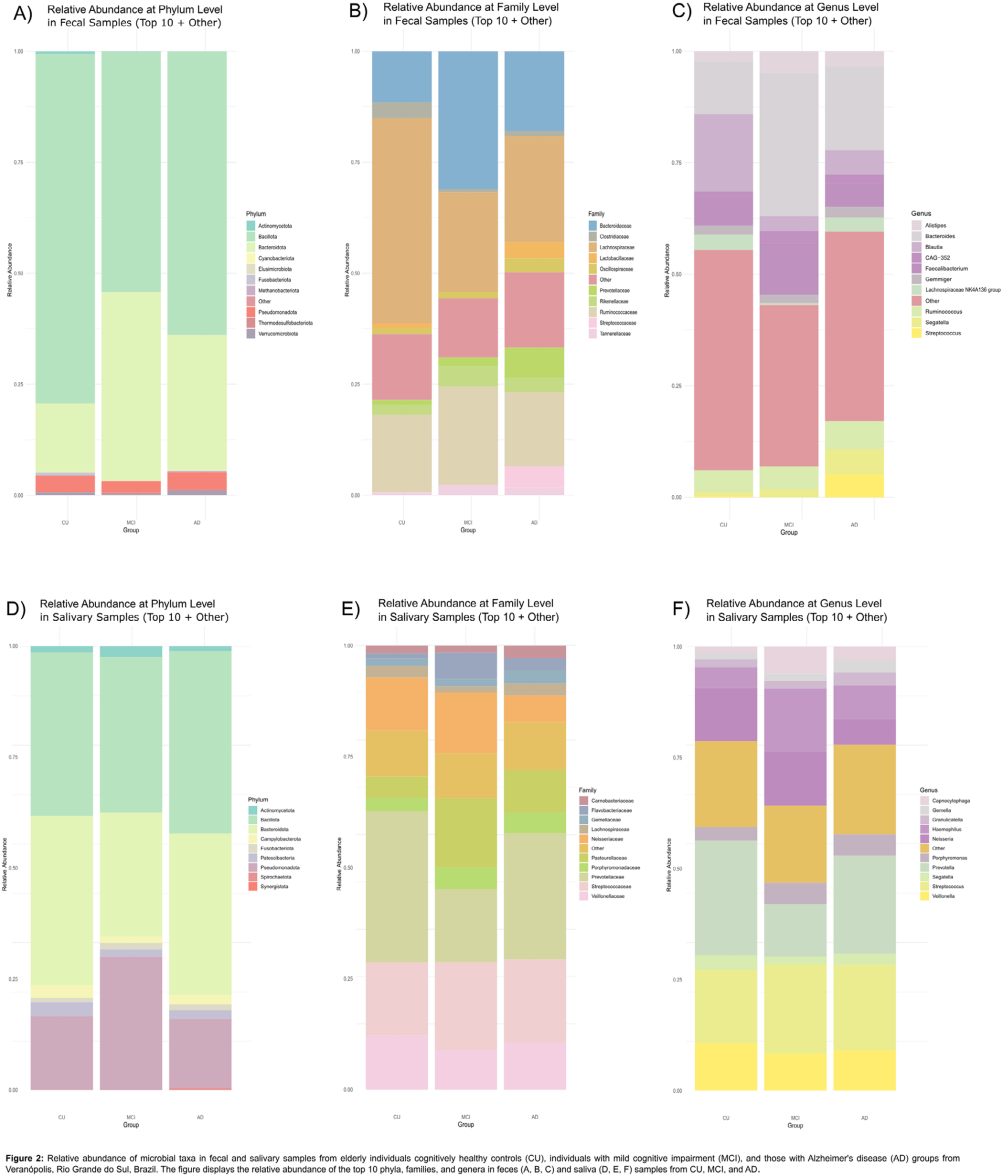# Oral and gut microbiota of elderly individuals with cognitive impairment in a Brazilian longevity hotspot

**DOI:** 10.1002/alz70855_106628

**Published:** 2025-12-24

**Authors:** Amanda Muliterno Domingues Lourenço de Lima, Marco De Bastiani, Wyllians Vendramini Borelli, Joana Emilia Senger, Berenice Maria Werle, Laisa Zanella, Lilian Vivian Netson, Ariele Detogni, Neide Maria Bruscato, Artur Francisco Schumacher‐Schuh, Joao Senger, Emilio Hideyuki Moriguchi, Lavinia Perquim, Isadora Crasnhak de Souza, Renan Antônio Barth, Eduarda Letícia Klafke Ebert, Rafaela Ramalho Guerra, João Vitor Cardoso Barboza, Eduardo José Gaio, Andreza Francisco Martins, João Batista Teixeira da Rocha, Diogo O. Souza, Eduardo R. Zimmer

**Affiliations:** ^1^ Universidade Federal do Rio Grande do Sul, Porto Alegre, Rio Grande do Sul, Brazil; ^2^ Universidade Federal do Rio Grande do Sul, Porto Alegre, RS, Brazil; ^3^ Instituto Moriguchi, Veranópolis, Rio Grande do Sul, Brazil; ^4^ Hospital de Clínicas de Porto Alegre, Porto Alegre, RS, Brazil; ^5^ Universidade Federal de Ciências da Saúde de Porto Alegre, Porto Alegre, Rio Grande do Sul, Brazil; ^6^ Hospital de Clínicas de Porto Alegre, Porto Alegre, Rio Grande do Sul, Brazil

## Abstract

**Background:**

Longevity is influenced by a combination of genetic factors, lifestyle choices, and environmental conditions. These factors can alter microbiota composition, potentially influencing susceptibility to Alzheimer's disease (AD) and cognitive decline. We hypothesize that the microbiome in elderly individuals may be associated with different clinical stages of AD. This study aims to investigate the relationship between oral and gut microbiota composition in a Brazilian long‐lived population and cognitive impairment within the AD continuum.

**Method:**

We conducted a pilot characterization of the oral and gut microbiota of 12 elderly individuals (>65 years) recruited by the Moriguchi Institute in Veranópolis, a longevity hotspot in southern Brazil. Participants underwent clinical‐cognitive assessment, including the Clinical Dementia Rating (CDR), and were classified as cognitively unimpaired (CU), mild cognitive impairment (MCI), or Alzheimer's disease (AD). Saliva and fecal samples were sequenced using Illumina MiSeq™, targeting the V3–V4 regions of the 16S rRNA gene, and processed in R using DADA2. Amplicon sequence variants (ASVs) were inferred, and taxonomic assignments were performed with SILVA. Abundance data were used for alpha and beta diversity, and relative abundance analyses.

**Result:**

Alpha diversity was similar across groups, except for reduced salivary richness in MCI (Chao1, *p* = 0.002; Figure 1). Rarefaction curves indicated higher richness in the feces compared to saliva. PCoA analysis showed distinct group separations in feces, with MCI and AD being more similar, while saliva samples were more uniform. Relative abundance demonstrated alterations in phylum Bacillota, Bacteroidota, and Pseudomonadota in MCI and AD compared to the CU group (Figure 2). Changes were particularly evident in fecal families Lachnospiraceae, Bacteroidaceae, and Ruminococcaceae, and genus such as *Bacteroides, Blautia*, and *Faecalibacterium*. Notably, *Streptococcus* was almost exclusively elevated in fecal samples of the AD group. Saliva samples were more homogeneous across groups, though changes were observed in families Prevotellaceae and Streptococcaceae, and genus *Prevotella, Streptococcus, Haemophilus*, and *Neisseria* in MCI and AD compared to CU.

**Conclusion:**

Fecal microbiota exhibited clinical‐stage‐specific changes, while salivary microbiota displayed more stability, underscoring microbial adaptations to the distinct. These findings highlight microbiome changes along the AD continuum, emphasizing the potential microbiome's role in healthy aging and resilience against neurodegeneration.